# CONNECT: Coordinating Options for Neurovascular Patients Needing Electrophysiology Consults and Treatments

**DOI:** 10.1177/19418744241289973

**Published:** 2024-09-29

**Authors:** Melissa Mortin, Ben Shifflett, Dawn M. Meyer, Lovella Hailey, Stephanie Yoakum, Jonathan C. Hsu, Brett C. Meyer

**Affiliations:** 1 Department of Neurosciences, 8784 University of California, San Diego, CA, USA; 2 Department of Cardiology, 8784 University of California, San Diego, CA, USA

**Keywords:** stroke, implantable loop recorders, electrophysiology, atrial fibrillation

## Abstract

**Background and Purpose:**

Though Event Monitors (EM) and Implantable Loop Recorders (ILR) are prevalent in stroke workups, complex processes to obtain placement of these device might result in delays. Our aim was to determine if the CONNECT (Coordinating Options for Neurovascular patients Needing Electrophysiology Consults and Treatments) pathway could improve Stroke-to-Electrophysiology (EP) communications, increase EM and ILR device placements prior to discharge, shorten placement time, and preserve satisfaction.

**Methods:**

We assessed device placements when an EP consult was obtained [Pre-CONNECT (5/1/21-4/30/22), CONNECT (5/1/22-4/30/23)] for patients with stroke. In the Pre- period, consults were sent via EPIC electronic medical record (EMR), with additional direct communication when desired. In the CONNECT period, the pathway and module allowed for immediate communication between services. Outcomes included case rate, times, length of stay, and satisfaction. Hospital reports detailed Order to Activation (O-A) days. EM report review was used to obtain O-A time. Clinician satisfaction was assessed using Qualtrics survey. Patient satisfaction was assessed with Hospital Consumer Assessment of Health care Clinicians and Systems (HCAHPS) survey. Man-Whitney U test was used.

**Results:**

78 patients were included (30EM(38.5%), 48ILR(61.5%)). Age was 68 years (*P* = 0.58). For ILRs, inpatient placements prior to discharge increased (3Pre vs 51 CONNECT; *P* < 0.0001) as did outpatient placements (5 vs 16; *P* = 0.02). Order to Activation (O-A) time savings were significant for ILR overall (32 days vs 1 day; *P* = 0.03) and for Inpatient EM (13 days vs 3 days; *P* = 0.003). Time for consultant to view was 4 min and to respond was 6 min. Devices were placed at a median 6 hrs 32 min (EM: 4 hrs 19 min; ILR:7 hrs36 min). All (12/12) clinicians preferred the technique. Patient satisfaction improved on 13/19 (68%) questions.

**Conclusions:**

There was a 1600% increase in ILR placements prior to discharge that was associated with the time period that the CONNECT process was in place. The robust improvement in ILR placements prior to discharge, high satisfaction, ease of use, closed loop communication, and respect for autonomy allowing more organic parallel discussions with patients improved clinician workflow which could potentially improve future risk reduction strategies.

## Introduction

Regarding assessments for Atrial Fibrillation (Afib) in stroke, some clinicians are including extended cardiac monitoring in their approach to stroke investigations, starting in the inpatient stroke evaluation period. Patients with stroke and Afib have improved outcomes when they are treated with anticoagulation therapy.^1-3^ Cryptogenic stroke population studies have revealed that Afib rates may be as high as 30% at 3 years.^4,5^ More recent data has extended this to a broader cohort of patients, showing an over 20% likelihood of Afib being found in 3 years even for those not historically felt to be cryptogenic.^6,7^ Telemetry and 24-hour Holter monitors have been used for arrhythmia detection, though over time the use of prolonged cardiac monitoring such as Event Monitors (EM) and Implantable Loop Recorders (ILRs) has become more common.

Non-streamlined processes may result in delay in initiating electrophysiology (EP) monitoring, which perhaps results in missing opportunities for earlier diagnosis and treatment. Viz. ai, along with other advanced Artificial Intelligence (AI) software companies, have developed platforms to help in the hyperacute assessment of patients with acute stroke. These tools include AI algorithms for assessing for large vessel occlusions (LVO), determining CT perfusion core/ penumbra mismatch, allowing mobile access to imaging, and enabling Health Information Portability and Accountability Act (HIPAA) compliant communication between stroke care teams such as Stroke and Neuro-Interventional (NIR) specialists. Platforms have now extended their capability to allow immediate communication for non-hyperacute stroke cases, and with other teams such as stroke, neuro-hospitalists, electrophysiology (EP) for ILR implantation, and Interventional Cardiology (IC) for Patent Foramen Ovale (PFO) closure. Our enterprise leveraged the Viz. ai tool to improve EP consult pathways for potential expedited placement of EM and ILRs prior to hospital discharge. Our aim was to determine if the newly deployed CONNECT (Coordinating Options for Neurovascular patients Needing Electrophysiology Consults and Treatments) pathway could result in improved numbers of ILR or EM device placements prior to hospital discharge. If this streamlined clinical workflow improved key performance indicators (KPIs) such as increased number of device placements, shorter time from consult request to placement (even prior to discharge), and preserved patient and clinician satisfaction, this could translate into improved care and streamlined risk reduction strategies for recurrent stroke.

## Methods

In this quality improvement (QI) initiative, we examined data from a single academic health center comprised of 2 comprehensive stroke centers (CSCs). Both the UCSD IRB (Project #805740) and the ACQUIRE Committee (Project #902) approved this project and determined that it is not regulated as human subjects research and thus does not require IRB approval. As a QI project, patients were not required to sign consent. This consecutive sample analysis included all inpatient acute stroke patients for whom an EP consult was obtained for purposes of EM or ILR placement. We compared the pre-CONNECT (5/1/21-4/30/22) to CONNECT (5/1/22-4/30/23) periods. In January 2021 we converted to using the Viz. ai platform for hyperacute NIR therapy for patients with LVOs. In 2022, the stroke team expanded its use to allow for potentially improved communication between the Stroke and EP services for patients in need of extended cardiac monitoring. We sought to assess if coordinated communication between Stroke and EP services would result in improved quantity of ILR or EM placements prior to hospital discharge. EM and ILR placement data from the outpatient setting was also analyzed.

Pre- CONNECT, standard pathways were used including sending EPIC EMR consult requests to the EP team, as well as using direct communication methods as clinicians felt indicated. Clinicians were able to place consults into EPIC, reach out directly to discuss cases with the EP consultant, and even send EPIC-Chat messages. In the CONNECT period, the stroke service clinicians utilized the Viz. ai module that was previously used for hyperacute strokes. Viz. ai’s HIPAA compliant communications tool allowed for addition of specific distribution groups for direct communication to the EP service line. As such, the stroke team was able to leverage this communications pathway for communications to EP regarding requests for cardiac device monitor placement prior to discharge.

The module was deployed to the stroke team (faculty, fellows, and nurse practitioners (NPs)), as well as the cardiac EP team (faculty and NP). Ten Stroke clinicians and 2 EP clinicians participated, and received a short training in use of the module. The module included radio button options to alert the consultant to type of device requested (EM or ILR), to enter clinically relevant variables, and to provide free text information about the patient with cryptogenic stroke. The stroke clinicians initiated communication to the EP team using the app when they determined there was an indication for EM or ILR, with this determination being left to the treating clinician based on their discussions with their patients. The EP team would get immediate notification on their smartphone device, and be able to communicate additional questions or provide details as to when the monitoring device could be placed. The device/ pathway was only utilized in the inpatient setting. There was no rigid policy imposed, with clinicians being free to request procedures when it was appropriate to do so. Similarly, EP clinicians were free to respond to these requests as they chose, without rigid rules or expectations regarding immediacy of reply. Our general principle is to request device placement (either EM or ILR) on the approximate last full inpatient hospital day to allow for the possibility that inpatient telemetry would find Afib during their hospitalization, thus limiting the need for EM or ILR if Afib were to be found. One device technique was not routinely encouraged over the other. Other than implementing the software module, and providing minimal education to the clinicians about the availability of this new communications tool, no other changes were made during the implementation period. Other than a short tutorial explaining the availability and use of the software module, no extended software training was performed.

KPIs included case rate, time to consultation, time to EM or ILR placement, actual analysis time, length of stay (LOS), and patient/ clinician satisfaction. Hospital reports were requested for patients who had ILRs or EMs placed to analyze number of placements in the Pre- and CONNECT periods. For “Order to Activation (O-A) time, hospital reports included “days from onset to service”. When 2 orders were placed for the same patient by the Stroke clinician, the first order was used. Missing hospital report data was completed by limited chart review for times. To obtain (O-A) time for EM cases, EM reports used time-stamp of EMR order for ordering time but limited chart review was required as activation time was documented in the monitoring report found in the chart. This also allowed an opportunity to document actual analysis times for EMs. In the CONNECT period the software tool was queried to collect age, site of referral, time consult was requested, viewed, responded to, and time consult was completed. Internal cost analyses were performed to assess financial metrics of the overall stroke population, the EM group, and ILR group in patients discharged with a stroke.

Clinician satisfaction with the CONNECT tool was assessed using a Qualtrics survey. Likert scale questions assessing both comparative and current satisfaction with the prior and new communication techniques were posed. Ten Stroke clinicians (requesting clinicians: 3 faculty physicians, 1 faculty NP, 4 stroke fellows, and 2 stroke NPs) and 2 EP clinicians (consultants: 1 faculty physician and 1 NP) were surveyed. Comparative questions included ease of use, speed of consult, and overall satisfaction. Questions assessing current satisfaction (ranging from “extremely satisfied” to “not satisfied at all”) were also posed for ease of use, speed of consult, and overall satisfaction. Patient satisfaction was assessed via Hospital Consumer Assessment of Health care Providers and Systems (HCAHPS) reports but were not specific to only the EM or ILR cohorts, but were filtered to only include relevant hospital ward locations where patients with stroke were cohorted. For statistical comparisons, Mann-Whitney U tests were used for non-parametric, non-evenly distributed data.

## Results

We identified 88 patients who had EP monitoring consult requests placed during the CONNECT period, with analysis done on 78 (5 with EM order and 5 with ILR order did not have device placed). Of the excluded 10 patients, reasons were well balanced (4 EM and 3 ILR patients refused device or deferred to potential outpatient discussion; 1 EM and 1 ILR patient had no reason documented, and 1 ILR patient had the request cancelled due to event not being a stroke). Final analysis included 78 patients including 30 EM (38.5%) and 48 ILR (61.5%). Average age was 68 years (EM = 69yo, ILR = 67yo; *P* = 0.58). Average length of hospital stay was 5 days in both groups. Consult request numbers differed between our hospital sites (32 (41%) from Site 1, 46 (59%) from Site (2) with more ILRs requested from our busier Site 2 (ILR: 18/48 (37.5%) from Site 1, 30/48 (62.5%) from Site 2; EM: 14/30 (47%) from Site 1, 16/30 (53%) from Site 2).

When assessing number of cardiac monitors (EM or ILR) placed prior to discharge, comparing Pre-CONNECT with CONNECT period, EMs showed little difference but ILRs were robustly increased (EM: 42 Pre vs 53 CONNECT; *P* = 0.26; ILR: 3 Pre vs 51 CONNECT; *P* < 0.0001). The outpatient setting also showed increased ILR (EM: 93 Pre vs 66 CONNECT; *P* = 0.03; ILR: 5 Pre vs 16 CONNECT; *P* = 0.02), though the increase was less robust. Overall, more ILRs were placed in the CONNECT time-period (ILR: 8 Pre vs 67 CONNECT; *P* < 0.0001). (Table 1)

**Table 1.** Case Numbers: ILR & EM (Pre vs CONNECT).

	Pre-CONNECT	CONNECT	*P *(Chi-squared)	Total N
Implantable Loop Recorders (ILR)				
Inpatient	3	51	<0.0001	54
Outpatient	5	16	0.02	21
Overall	8	67	<0.0001	75
Event monitors (EM)				
Inpatient	42	53	0.26	95
Outpatient	93	66	0.03	159
Overall	135	119	0.32	254
Combined devices (ILR + EM)				
Inpatient	45	104	<0.0001	149
Outpatient	98	82	0.23	180
Overall	143	186	0.02	329

Case numbers for Implantable Loop Recorder (ILR) and external Event Monitor (EM) during the Pre-CONNECT and CONNECT implementation periods. Inpatient, Outpatient, and Overall total number of devices placed are listed, as are related *P*-values.

Table 2 shows the improved time from device Order to Activation (O-A) in the CONNECT period. When using hospital provided reports to determine O-A times, ILRs ordered prior to discharge were performed quickly (Inpatient ILR: 1 day Pre vs 0 days CONNECT; *P* = 0.53) but did not differ statistically (though there were only 3 cases of ILR placement total in the Pre- period). There was no difference in Outpatient (ILR: 51 days vs 65.5 days; *P* = 0.97) but there was a difference overall (Inpatient + Outpatient) (ILR: 31.5 days vs 1.0 days; *P* = 0.03) which was driven by the robustly short time to place ILRs prior to discharge. Combined, any devices (EM + ILR) requested to be placed prior to discharge required only 6 hrs 32 min (EM: 4h rs 19 min; ILR: 7 hrs 36 min). In the CONNECT period, time to consultant viewing the request was only 4 min, and time to consultant response was only 6 min. When using hospital provided O-A time reports for EMs ordered prior to discharge, times improved significantly (Inpatient EM: 13.0 days Pre vs 2.5 days CONNECT; *P* = 0.003). In the CONNECT period, subset analysis showed a significant difference favoring the scenario when the CONNECT module was used (12 days when it was not used, 0 days when it was used; *P* < 0.0001). There was also a difference noted in outpatient EM placement (EM: 27 days vs 11.5 days; *P* < 0.0001) and overall (Inpatient + Outpatient) (EM: 22 days vs 9.0 days; *P* < 0.0001).

**Table 2.** ‘Time Ordered to Time Activated (O-A)’ Times: Median Days (Pre vs CONNECT).

	Pre-	CONNECT	*P*
Implantable Loop Recorders (ILR)			
Inpatient	1.0 days	0.0 days	0.53
Inpatient (hours)		7 hrs 36 min	
Outpatient	51.0 days	65.5 days	0.97
Overall	31.5 days	1.0 days	0.03
Event monitors (EM)			
Inpatient	13.0 days	2.5 days	0.0003
		*Not used **CONNECT used	
		12.0 0.0	<0.0001
Inpatient (hours)		4 hrs 19 min	
Outpatient	27.0 days	11.5 days	<0.0001
Overall	22.0 days	9.0 days	<0.0001
Combined devices (ILR + EM)			
Inpatient (hours)		6 hrs 32 min	

Time from ordering device until time device is placed/ activated. This (O-A) time is reported for both Implantable Loop Recorders (ILR) and external Event Monitors (EM) during the Pre-CONNECT and CONNECT implementation periods. Inpatient, Outpatient, and Overall totals are listed, as are related *P*-values. Overall data drawn from hospital reports is shown in “days to device placement/ activation”. Limited chart review was done, to report <1-day values in hours/minutes. A subset assessment of days until EM device was placed is also shown for cases where the CONNECT module was used* and when it was not used**.

In our institution clinicians can only order either 14 day or 30-day EM monitoring. Table 3 notes no differences in median length of time EMs were ordered for the inpatient setting (EM: 29.0 median days Pre and 29.0 days CONNECT; *P* = 0.25), for the outpatient setting (EM: 14.0 days Pre vs 17.5 days CONNECT; *P* = 0.43), or for the overall (Inpatient + Outpatient) EM population (EM: 16.0 days Pre vs 29.0 days CONNECT; *P* = 0.05). Chart review done to assess actual monitoring time showed no differences (EM: 14 days Pre vs 14 days CONNECT; *P* = 0.58). An internal cost analysis snapshot was performed based on period specific financial metrics for the overall stroke population as well as both the ILR and EM groups. ILR placements were financially sustainable.

**Table 3.** Event Monitor (EM) Times: (Pre vs CONNECT) in Median Days.

	Pre-CONNECT	CONNECT	*P*
Event Monitors (EM): Time Device Ordered for			
Inpatient	29.0 (60% 30d)	29.0 (80% 30d)	0.25
Outpatient	14.0 (40% 30d)	17.5 (50% 30d)	0.43
Overall	16.0 (52% 30d)	29.0 (62% 30d)	0.05
Event monitors (EM): Time of analysis			
Inpatient	14.0	14.0	0.81
Outpatient	14.0	14.0	0.71
Overall	14.0	14.0	0.58

Length of time that clinicians ordered Event Monitors (EM) during the Pre-CONNECT and CONNECT implementation periods. Inpatient, Outpatient, and Overall totals are listed, as are related *P*-values. Percentage values are % of EM devices ordered for a 30-day monitoring period instead of a 14-day period. Also shown is the actual “analysis” time drawn from EM reports.

When comparing the prior pathway to the new CONNECT pathway, 100% of the clinicians responded to the survey. All 12/12 (100%) clinicians preferred the new technique on “Ease” 12/12 (100%), “Speed” 12/12 (100%), and “Overall” 12/12 (100%). For questions focusing on clinicians’ current opinion of the new technique, 12/12 (100%) clinicians reported being “Extremely Satisfied” with Viz-Connect for all 3 measures. When assessing HCAHPS data, compared to the Pre-CONNECT time-period, there was improvement on 13/19 (68%) questions related to the 9 dimensions of care. In the CONNECT period, 15/19 (79%) questions were above benchmark comparison, which increased from 13/19 (68%) from the Pre- period. Net Promotor Score (NPS) question regarding likelihood to recommend improved from 79.7% to 82.4%. Survey response rate was 17.9% Pre vs 21.6% during CONNECT.

## Discussion

Though the overall US stroke population averages 74 years old, cryptogenic stroke patients are younger.^8-10^ Our cohort was older than the classic cryptogenic stroke population showing that more older patients may be receiving extended cardiac monitoring. Age was not different between EM and ILR recipients, signaling that device choice was not based on age.

**Figure 1.**
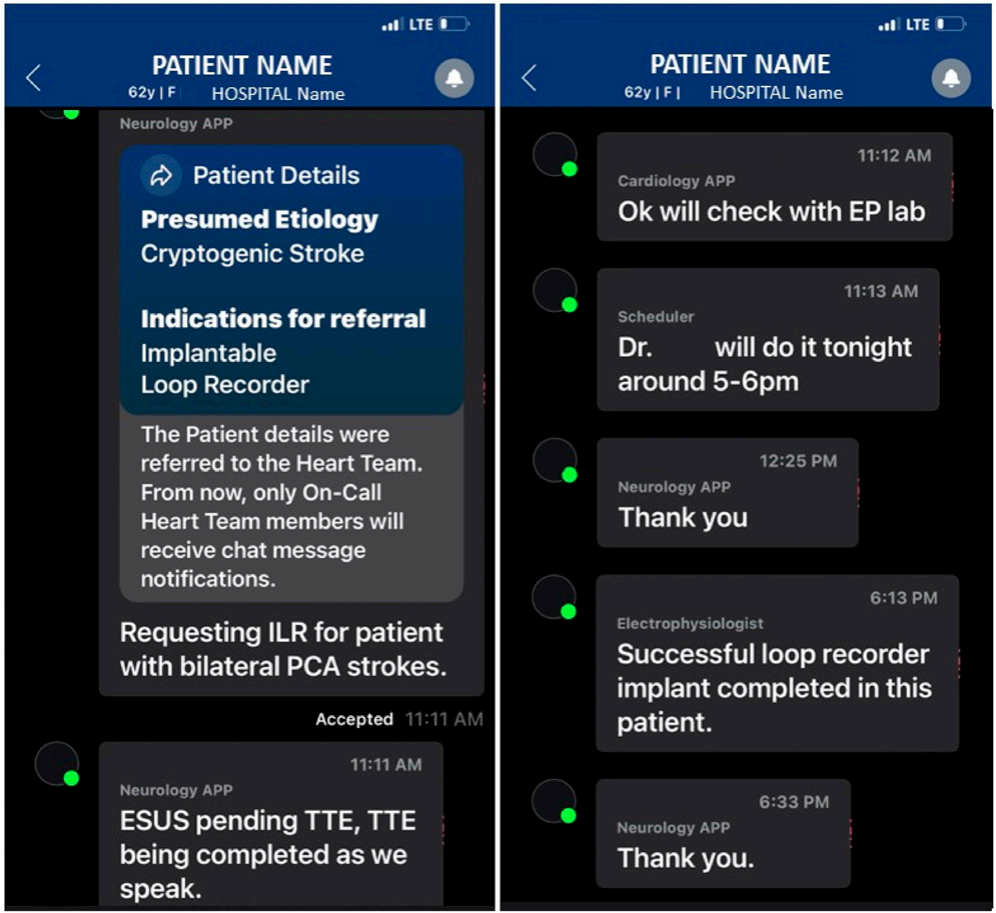
Sample CONNECT Communication. Inter-disciplinary team communication regarding a request for Implantable Loop Recorder (ILR) implantation. The Stroke team clinician accesses the CONNECT module, enters minimal data into standard fields, and may also add-in free text information about the patient case. This results in HIPAA complaint, real-time notification of the electrophysiology (EP) team and subsequent communication pathway to streamline ILR (or event monitor) placements. This facilitates timely device implantation (7 hrs 22 min in this case) and allows for EP specialist to be able to communicate back to the requesting Stroke team, in real-time, to close the loop of communication.

This CONNECT pathway was created to assess for possible increased numbers of advanced cardiac monitors placed which included both EM and ILR devices. We noted a 1600% increase in ILR placements prior to hospital discharge that was associated with the time period that the CONNECT process was in place, and perhaps due to the availability of this HIPAA compliant, multi-team, communications tool. Our institution has used EPIC for many years. Prior to this new technique, our standard practice was to place EPIC consults with subsequent direct communication which included options such as EPIC-Chat. Though these pathways allowed for paging, texting, and even direct messaging to the EP team, for some reason, it did not translate into using it to obtain more EM or ILR placements. This could be due to clinicians not wanting EPIC-Chat notifications on their handheld devices for all hospital purposes, but instead desiring direct communication between only these 2 teams (Stroke and EP Cardiology). The old system was complex, resulting in few (n = 3) ILRs being placed prior to discharge. This new pathway with availability of alerts, alarms, and immediate access on handheld through the Viz. ai CONNECT platform simplified the process for our clinicians allowing immediate interaction between the teams. Being able to immediately notify the EP clinician of the need for placement, obtaining near-immediate response, and getting feedback upon completion of the ILR placement was invaluable to our processes. (Figure 1). Similarly, the placement of ILRs prior to discharge instead of placing them as outpatient many months later also allowed for hospital focused advanced risk factor management. Increasing device placements has significance as the incidence of Afib is prominent at 5%–6% per 1000 person-years and prevalence is increasing in the US population.^11,12^ This new communication process did not simply “left-shift” the number of ILR placements from the outpatient to the inpatient setting. There was also an increase seen in the outpatient setting. (5 Pre vs 16 CONNECT). Finding a streamlined process to enable early EM or ILR placement might therefore result in quicker assessments to find arrhythmias earlier.

We believe we noted an increase in ILR placements more so than EM placements for multiple reasons. First, given the difference between number of ILRs placed prior to discharge in the pre vs post period, it is clear that availability made much of the difference. Clinicians likely desired to have these ILRs placed in the past, but there was no carved-out pathway to contact EP, discuss the need, and implant the device in the pre-period. The simple availability of the option and process drove the increased placements. Second, the availability of what we have termed “CONNECT-Organic”, also drove the increase in ILRs more than EMs. Starting with a non-invasive test before pursuing a more invasive alternative is a foundational principle applicable to many areas of clinical practice. However, “respect for patient autonomy” is also a fundamental ethical principle which allows patients to choose a non-invasive monitor over an invasive monitor (due to less procedural risk), or an invasive monitor over a non-invasive one so that it can provide data for far longer than 30 days (due to longer term monitoring and subsequent simplicity) should they choose. Patients were offered a parallel choice prior to discharge, and our results showed that 62% of the time, patients preferred going straight to ILR placement.

Devices were also placed far faster in the CONNECT period (Table 2). Overall, ILRs were placed in <1 day (7 hrs36 min) for CONNECT vs requiring a month in the Pre- period. The inpatient ILR data is interesting because although there were only 3 inpatient ILRs placed in the Pre- period, the median time to device placement was only 1 day, showing that if we were to develop a system (such as CONNECT) where ILR needs could be quickly communicated, it was possible to get them placed before discharge. Even for EMs, whether in inpatient (13 days vs 2.5 days) or outpatient (27 days vs 11.5 days), the CONNECT period was faster. This shows that the CONNECT pathway worked well for both non-invasive EM monitoring and for ILRs.

The time improvement noted was linked to actual CONNECT module use. Subset analysis showed 0 days for device placement when the module was used compared to 12 days when it was not used. Thus, when CONNECT was used, EMs were placed far quicker. It wasn’t just due to overall awareness of monitoring processes, an educational trend that patients need cardiac monitoring, or awareness of the CONNECT program that caused the improvement. In fact, the CONNECT communications tool/ pathway itself contributed to the difference in times.

One of the reasons patients don’t seek medical care is because “real-life” responsibilities cause patients to be too busy to obtain follow up care.^13^ Our concern was that the longer it takes to obtain EP monitoring, the less likely that patients will receive that monitoring. It also may simply be easier to place an EM prior to beginning a more complicated referral process to obtain an ILR (which may never get done). This real-world barrier to ILR placement may not be uncommon, and may result in simply choosing the “easier” choice.

This ILR to EM ratio was not the distribution prior to the start of this CONNECT pathway. In the Pre- period, our predominant option was to schedule outpatient device placement (usually EMs first for simplicity). Our center mostly performed EMs because there was not a quick option for ILR placement. Although there was an intention to perhaps do ILRs after the EM was completed, our results show that this did not happen (as there were only 5 ILRs placed in the outpatient setting in the Pre- period).

It may be questioned whether the increased ILR placements prior to hospital discharge was due to CONNECT or more due to the trend of clinicians simply doing more EMs and ILRs. Our results point to the CONNECT module being critically important to the improved device placements. First, if this finding were simply due to a national trend of doing more monitoring, our rate of outpatient ILRs should have increased to the same degree. Second, the number of EMs placed in the outpatient setting actually decreased over time. Third, our subset analysis of EM placement comparing when CONNECT module was used to when it was not used (in the same time-period) showed a module- specific difference in favor of CONNECT (0 days vs 12 days) which would also not be expected if published literature were driving these placements.

Table 3 shows that when EMs were ordered in the inpatient setting, they were generally ordered for a 30-day period (with 52% ordered for 30 days in the Pre-period vs 62% ordered for 30 days in the CONNECT period). Although speculative, this may be because if EMs were ordered as an inpatient the clinician may have felt the case was significant enough to warrant the full 4-week monitoring time. Our “On-analysis” time result supports that early placement of these devices reassuringly did result in patients continuing to wear these devices to the same degree as they otherwise would have.

The 2-way, synchronous communication tool was felt to have both high ease of use, speed of use, and overall provider satisfaction. Similarly, patient satisfaction was high. Although it was beyond the scope of this QI project to send out CONNECT specific surveys, we were able to assess standard HCAHPS surveys, filtered to the appropriate time periods and hospital ward locations where our patients with stroke were cared for. We noted preserved to improved satisfaction over time. The greatest patient-facing benefit of the CONNECT technique was not anticipated nor planned, and would not be noted in HCAHPS surveys. Historically, owing to not yet having a streamlined process to obtain ILRs prior to discharge, we were never previously able to simultaneously offer either EMs or ILRs prior to discharge. This resulted in a serial plan of care which usually included the provider ordering an EM placement only, then perhaps having the clinician consider an ILR as outpatient. This choice for the patient (due to perceived limited availability of placing ILRs quickly) does not support the principle of “respect for patient autonomy”.^14,15^ During CONNECT, clinicians noted a more organic ability to communicate the option for either EM or ILR directly with patients. A more organic discussion with the patient allowed the clinician to offer both options to the patient, discuss risks and benefits, and allow the patient to determine if they wanted the non-invasive but perhaps more complicated process (sometimes having to take the patch on and off, and obtaining only 14-30 days of data) vs a more-invasive option (but less maintenance required post implantation of the ILR). Patients were able to contribute to their own decision-making by interacting directly with their clinician in these discussions (the patients did not interact with the CONNECT module itself). In 62% of these cases, patients opted for direct implantation of the ILR. This respect for patient autonomy can-not be over-stated.

Our study has limitations, most of which are due to the QI approval which limited the design. There were 10 patients who were excluded due to not receiving their EM or ILR. These patients were evenly split between EM and ILR, and reasons were well balanced. We also did not compare the baseline characteristics in Pre- and CONNECT, or between our 2 CSCs. However, the equal patient age and equal length of stay between EM and ILR groups, point to balance in baseline characteristics. As there were only 3 ILR placements in the pre-period, comparisons of baseline characteristics would not be meaningful. Our hospital system is split into two facilities, located ∼11 miles from each other and serving the same population. They operate as one facility, using the same EMR, and with the same service lines covering them. Clinicians at either institution might be the clinicians placing the ILR. However, we cannot completely exclude some unknown bias based on facility. A larger subsequent sample size will help ensure validity of our results.

The availability of placing ILRs prior to discharge did allow the clinicians to take advantage of that option. The authors cannot say that results of recent publications emphasizing the yield of ILR monitoring, and perhaps even the small amount of additional messaging and education surrounding this initiative, did not play a role in increasing the number of and speed of device placements.^4-7^ However if causative, these educational discussions would have been expected to increase both the inpatient and outpatient ILR placement numbers, which was not seen. It is possible that a pathway built around placing the EPIC order, and directly text messaging the EP provider, could have resulted in success as well. In that case, other hurdles such as always knowing which EP provider is on at any given time, ensuring providers are using EPIC-chat, and mitigating against using other non-HIPAA compliant text-messaging pathways, would have to be implemented as well. However, pathways such as this could be developed and could potentially result in success. The possibility must also be noted that a systematic campaign encouraging stroke and EP clinicians to use Epic-chat instead of Viz. ai CONNECT could have allowed for the communication portion to take place (though all other alerts and pick-lists would not have been available). There may have been performance or measurement bias accounting for the differences in total number of devices placed during the pre-CONNECT vs CONNECT periods, due to provider knowledge that communications would be monitored. The continued placement of >90 ILRs in the subsequent 12-month period makes this less likely but this is still a possibility. Also, assessing whether early ILR placement provides clinically relevant data in a way that other less invasive options cannot, is not definitively proven by this analysis. Published data suggests the average time to find Afib on these monitors is many months, perhaps limiting the yield of a 14-30-day EM. We do note that giving patients a choice for a non-invasive EM or more invasive ILR device placement is ethically important for “respect for patient autonomy”. Also, since comparison of clinical outcomes was beyond the scope of this analysis, it is important to note that the greater quantity of ILR or EM placement prior to hospital discharge, as seen in this CONNECT study, does not mean that implantation of more ILRs is better, earlier device placement leads to earlier detection of Afib, or earlier detection of Afib results in improved outcomes. Placing more ILRs to find more Afib may not be better to improve long term outcome. This process just allows providers and patients the choice to obtain either EMs or ILRs prior to discharge. Finally, whether the high case rate will be sustained long term has not yet been verified, but preliminary findings show sustainability. We performed approximate 50 ILR cases per year in this analysis, while our present rolling 12-month average is > 90 cases per year.

CONNECT resulted in an improved operational workflow and increased ILR placements prior to discharge. The robust improvement in numbers of ILR placements prior to hospital discharge, coupled with high satisfaction, ease of use, the critical importance of closed loop communication in health care teams, and respect for autonomy allowing more organic parallel discussions with patients, have improved clinician workflow. This may translate into improved risk reduction strategies to reduce recurrent stroke. Widespread utilization of the CONNECT pathway could help increase monitoring opportunities for patients and help identify a greater number of patients with Afib.

## Data Availability

Original data is available upon request.*

## References

[1] Predictors of thromboembolism in atrial fibrillation: I. Clinical features of patients at risk. The Stroke Prevention in Atrial Fibrillation Investigators. Ann Intern Med. 1992;116(1):1-5. doi:10.7326/0003-4819-116-1-1 1727091

[2] Predictors of thromboembolism in atrial fibrillation: II. Echocardiographic features of patients at risk. The Stroke Prevention in Atrial Fibrillation Investigators. Ann Intern Med. 1992;116(1):6-12. doi:10.7326/0003-4819-116-1-6 1727097

[3] Adjusted-dose warfarin versus low-intensity, fixed-dose warfarin plus aspirin for high-risk patients with atrial fibrillation: stroke Prevention in Atrial Fibrillation III randomised clinical trial. Lancet. 1996;348(9028):633-638. 8782752

[4] Sanna T Diener HC Passman RS , et al. Cryptogenic stroke and underlying atrial fibrillation. N Engl J Med. 2014;370(26):2478-2486. doi:10.1056/NEJMoa1313600 24963567

[5] Brachmann J Morillo CA Sanna T , et al. Uncovering atrial fibrillation beyond short-term monitoring in cryptogenic stroke patients: three-year results from the cryptogenic stroke and underlying atrial fibrillation trial. Circ Arrhythm Electrophysiol. 2016;9(1):e003333. doi:10.1161/circep.115.003333 26763225

[6] Bernstein RA Kamel H Granger CB , et al. Effect of long-term continuous cardiac monitoring vs usual care on detection of atrial fibrillation in patients with stroke attributed to large- or small-vessel disease: the STROKE-AF randomized clinical trial. JAMA. 2021;325(21):2169-2177. doi:10.1001/jama.2021.6470 34061145 PMC8170544

[7] Bernstein RA Kamel H Granger CB , et al. Atrial fibrillation in patients with stroke attributed to large- or small-vessel disease: 3-year results from the STROKE AF randomized clinical trial. JAMA Neurol. 2023;80:1277-1283. doi:10.1001/jamaneurol.2023.3931 37902733 PMC10616765

[8] Akyea RK Vinogradova Y Qureshi N , et al. Sex, age, and socioeconomic differences in nonfatal stroke incidence and subsequent major adverse outcomes. Stroke. 2021;52(2):396-405. doi:10.1161/strokeaha.120.031659 33493066 PMC7834661

[9] Hart RG Diener HC Coutts SB , et al. Embolic strokes of undetermined source: the case for a new clinical construct. Lancet Neurol. 2014;13(4):429-438. doi:10.1016/s1474-4422(13)70310-7 24646875

[10] Diener HC Easton JD Hart RG Kasner S Kamel H Ntaios G . Review and update of the concept of embolic stroke of undetermined source. Nat Rev Neurol. 2022;18(8):455-465. doi:10.1038/s41582-022-00663-4 35538232

[11] Williams BA Chamberlain AM Blankenship JC Hylek EM Voyce S . Trends in atrial fibrillation incidence rates within an integrated health care delivery system, 2006 to 2018. JAMA Netw Open. 2020;3(8):e2014874. doi:10.1001/jamanetworkopen.2020.14874 32857147 PMC7455855

[12] Colilla S Crow A Petkun W Singer DE Simon T Liu X . Estimates of current and future incidence and prevalence of atrial fibrillation in the U.S. adult population. Am J Cardiol. 2013;112(8):1142-1147. doi:10.1016/j.amjcard.2013.05.063 23831166

[13] Kullgren JT McLaughlin CG Mitra N Armstrong K . Nonfinancial barriers and access to care for U.S. adults. Health Serv Res. 2012;47(1 Pt 2):462-485. doi:10.1111/j.1475-6773.2011.01308.x 22092449 PMC3393009

[14] Varelius J . The value of autonomy in medical ethics. Med Health Care Philos. 2006;9(3):377-388. doi:10.1007/s11019-006-9000-z 17033883 PMC2780686

[15] McGehrin K Spokoyny I Meyer BC Agrawal K . The COAST stroke advance directive: a novel approach to preserving patient autonomy. Neurol Clin Pract. 2018;8(6):521-526. doi:10.1212/cpj.0000000000000549 30588382 PMC6294541

